# CDX2 Expression and Fluoropyrimidine Response in Rare Non-GI Tumors: A Three-Case Series

**DOI:** 10.3390/curroncol33020126

**Published:** 2026-02-21

**Authors:** Riham Suleiman, Andrea Dipp Garcia, Binav Baral, Thorvardur Halfdanarson, Harry Fuentes-Bayne

**Affiliations:** 1 Department of Oncology, Mayo Clinic, 200 First Street SW, Rochester, MN 55905, USA; suleiman.riham@mayo.edu (R.S.); baral.binav@mayo.edu (B.B.); halfdanarson.thor@mayo.edu (T.H.); 2 School of Medicine, Universidad Autónoma de Guadalajara, Av. Patria 1201, Lomas del Valle, Zapopan C.P. 45129, Jalisco, Mexico; andreadipp0726@gmail.com

**Keywords:** CDX2, fluoropyrimidine, rare cancers, phenotype-driven therapy, immunohistochemistry

## Abstract

Caudal type homeobox 2 (CDX2) is an intestine-specific transcription factor that serves as a diagnostic marker of enteric differentiation and may also reflect tumor behavior and therapeutic susceptibility. Emerging evidence suggests that CDX2 expression may predict sensitivity to fluoropyrimidine-based therapy independent of tissue of origin. We report a retrospective case series of three patients with metastatic adenocarcinoma (aggressive variant prostate, minor salivary gland, and intestinal-type sinonasal tract) exhibiting strong CDX2 nuclear expression. In all cases, tumors were refractory to or lacked established standard systemic therapy. Treatment decisions were informed by the CDX2-positive enteric phenotype, leading to the initiation of fluoropyrimidine-based regimens. Response was assessed using PET-CT and MRI. All three patients achieved marked metabolic and clinical responses, including a sustained complete metabolic response in the prostate cancer case and durable disease control in the salivary gland and sinonasal tumors. These findings highlight CDX2 as a potential biomarker requiring validation, which may identify tumors intrinsically susceptible to fluoropyrimidines regardless of anatomical origin. CDX2 immunohistochemistry is widely available and inexpensive, and may complement genomic profiling in rare malignancies or in settings where standard treatment algorithms are limited. This report is hypothesis-generating and not intended to estimate response rates or treatment efficacy.

## Simple Summary

CDX2 is a protein normally expressed in intestinal cells and is widely used by pathologists to help determine the origin of tumors. However, CDX2 may also carry important therapeutic information. In this study, we describe three patients with rare metastatic cancers of the prostate, salivary gland, and sinonasal tract whose tumors strongly expressed CDX2, indicating an intestinal-like phenotype. Standard treatments were ineffective or not well established in these cases. Based on CDX2 expression, patients were treated with chemotherapy typically used for colorectal cancer. All three experienced substantial clinical and radiologic improvement. These cases suggest that CDX2 may serve not only as a diagnostic marker but also as a potential biomarker warranting further evaluation for treatment selection across different cancer types, particularly in rare tumors where evidence-based systemic options are limited.

## 1. Introduction

Caudal type homeobox 2 (CDX2) is an intestine-specific nuclear transcription factor encoded by the human homolog of the *Drosophila* caudal homeobox gene [1]. It plays a central role in establishing and maintaining intestinal epithelial identity during embryonic development and throughout adulthood [2]. Experimental models have shown that CDX2 is indispensable for gut morphogenesis, the maintenance of the intestinal epithelial phenotype [3], activation of intestine-specific genes [4], expression of differentiated intestinal enzymes [4,5], and regulation of cellular proliferation [5,6]. In normal human tissues, CDX2 expression is localized to epithelial nuclei of the small intestine, colon, and appendix [7], with additional expression in pancreatic ductal and centroacinar cells [8]. Ectopic expression in gastric mucosa can induce intestinal metaplasia, underscoring its ability to drive enteric differentiation beyond the gastrointestinal (GI) tract [9,10,11].

In malignancy, CDX2 is most prominently expressed in colorectal adenocarcinomas, where more than 90% of cases show nuclear positivity [12], establishing it as a sensitive diagnostic marker of colorectal origin [13,14]. However, CDX2 expression is also found in gastric [15,16] and pancreatobiliary adenocarcinomas [17], and even in tumors arising outside the GI tract, including those of the sinonasal tract [18], lung [19,20], ovary [21], prostate [22], and bladder [23]. This distribution indicates that CDX2 reflects a broader enteric differentiation program rather than a strictly site-specific phenotype.

Growing evidence further suggests that CDX2 expression may be associated with tumor behavior and therapeutic susceptibility [21,24,25]. CDX2-positive tumors retain enterocyte-like programs, including pathways relevant to fluoropyrimidine response. In our recent CDX2-positive cancer of unknown primary (CUP) cohort, fluoropyrimidine-based therapy produced higher response rates and longer progression-free and overall survival than non-fluoropyrimidine regimens, independent of site-suggestive immunophenotypes [26]. These findings suggest that CDX2 may represent a phenotype-driven biomarker identifying tumors intrinsically sensitive to fluoropyrimidines and less responsive to taxanes, regardless of anatomic origin. Here, we describe three cases of metastatic adenocarcinoma of the prostate, salivary gland, and sinonasal tract in which CDX2 expression suggested enteric differentiation and informed fluoropyrimidine-based treatment. These cases underscore the growing diagnostic and predictive significance of CDX2 across diverse primary sites.

## 2. Case Presentation

### 2.1. Case 1

A 75-year-old never-smoker male patient with a history of high-risk prostate cancer treated with SBRT and leuprolide presented in January 2023 with a rising PSA of 3.9. CT scan showed a presacral mass and bilateral pulmonary nodules. Given radiologic progression of his prostate cancer, he started docetaxel; however, subsequent imaging demonstrated further progression involving the presacral lesion, pelvic lymph nodes, and pulmonary nodules.

**Figure 1:**
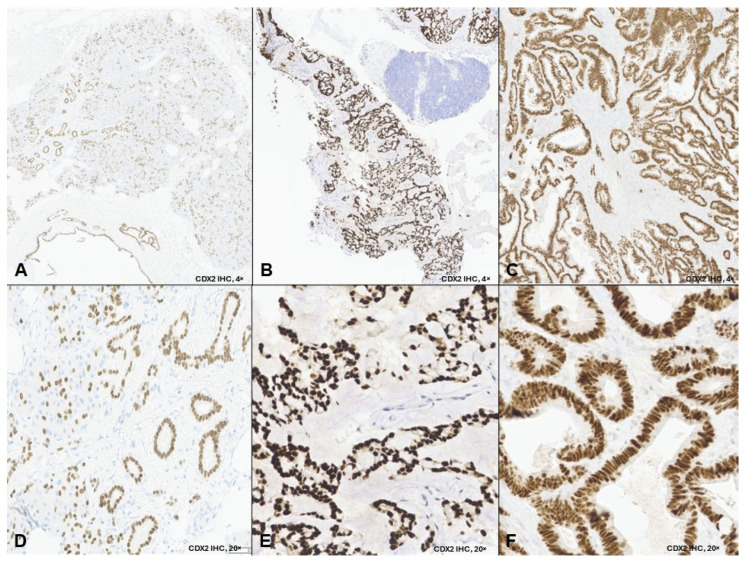
CDX2 immunohistochemistry in three non-gastrointestinal adenocarcinomas. Legend: Representative low-power (4×; (**A**–**C**)) and high-power (20×; (**D**–**F**)) images demonstrate nuclear CDX2 expression in tumor cells from: Case 1, lung biopsy showing metastatic carcinoma with patchy strong nuclear CDX2 positivity (**A**,**D**); Case 2, cervical lymph node biopsy showing metastatic adenocarcinoma with enteric differentiation and diffuse strong nuclear CDX2 expression (**B**,**E**); and Case 3, nasal mass biopsy showing intestinal-type adenocarcinoma with diffuse strong nuclear CDX2 expression (**C**,**F**). Low-power images illustrate the distribution of CDX2-positive tumor cells, while high-power images highlight nuclear staining specificity.

A biopsy of the pulmonary nodules revealed a poorly differentiated carcinoma positive for AE1/AE3, CK7, p16, and SATB2 with patchy strong nuclear staining for CDX2 (Figure 1A,D). Tumor cells were faintly positive for NKX3.1, synaptophysin, and INSM1, with a Ki-67 index > 90%. The tumor was negative for TTF-1, CK20, p40, PSA, PSAP, AR, ERG, chromogranin, GATA3, uroplakin II, and SOX10. Molecular testing demonstrated microsatellite stability, a tumor mutational burden of 0, and copy number loss of RAD51C, PTEN, and TBL1XR1. No common colorectal cancer-associated mutations were detected.

**Figure 2:**
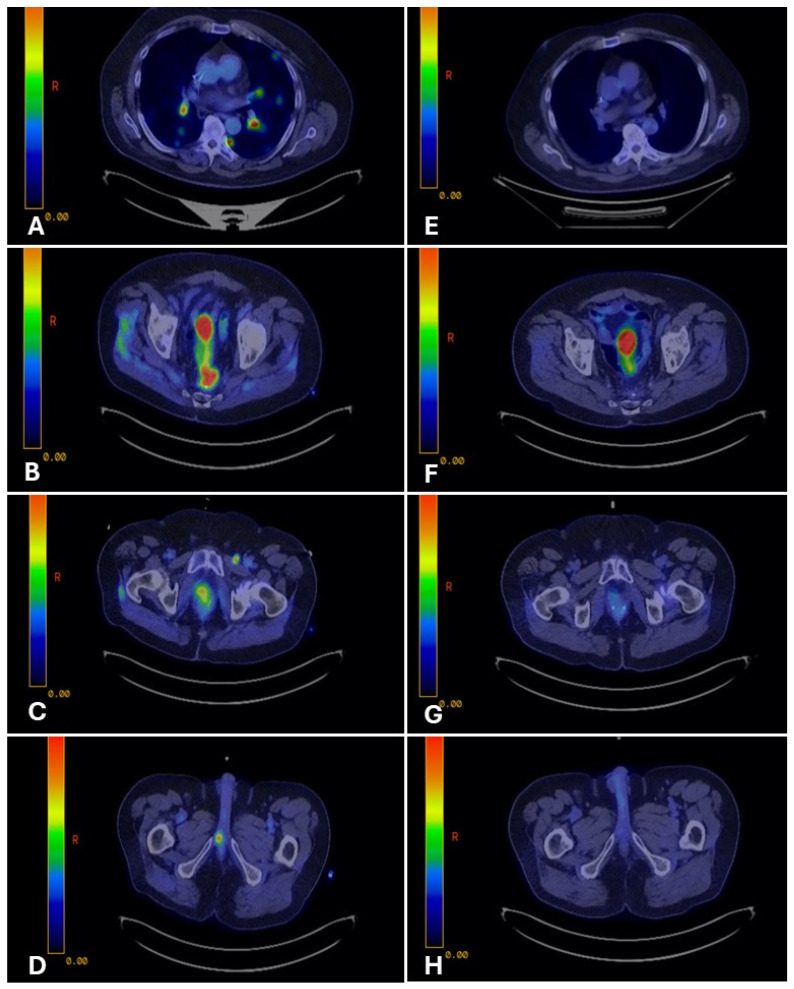
PET-CT imaging before and after fluoropyrimidine-based therapy in Case 1. Legend: Representative PET-CT images demonstrate FDG-avid disease at multiple sites before therapy and the response after fluoropyrimidine-based treatment. Pre-treatment (Panels (**A**–**D**)): FDG-avid disease involving bilateral lungs (**A**), presacral pelvic mass (**B**), pelvic lymph nodes (**C**), and right penile crus (**D**). Post-treatment (Panels (**E**–**H**)): Restaging PET-CT images show complete metabolic response at all previously involved sites, with resolution of FDG uptake (**E**–**H**).

PET-CT on April 5, 2023, confirmed FDG-avid disease in the presacral region, bilateral lungs, left inguinal and right iliac chain lymph nodes, and right penile crus (Figure 2A–D). PSMA-PET revealed a concordant uptake in the same regions. The patient was subsequently evaluated in the CUP clinic at Mayo Clinic. The overall findings, including the patient’s history, persistent NKX3.1 expression, and diffuse PSMA uptake, favored a dedifferentiated prostate carcinoma with divergent enteric morphology. Given CDX2 positivity and prior progression on docetaxel, a histology-driven approach was adopted using FOLFOX (leucovorin, 5-fluorouracil [5-FU], and oxaliplatin). After six cycles, FDG PET-CT demonstrated a complete metabolic response (Figure 2E–H**)**, sustained on the 3-month restaging scan. He was subsequently maintained on 5-FU and leucovorin.

Four months after initiating maintenance therapy, he developed imbalance; brain MRI revealed multiple enhancing lesions. The dominant lesion was resected and followed by whole-brain radiation, confirming metastatic adenocarcinoma with identical morphology. Extracranial disease remained in complete response.

Given concern for CNS sanctuary progression, irinotecan was added to 5-FU due to its superior CNS penetration, and PET-CT three months later again showed no FDG-avid disease. As his performance status declined, systemic therapy was transitioned to olaparib monotherapy based on the RAD51C mutation, with capecitabine reserved for later use if progression occurred. Serial MRI and PET-CT demonstrated ongoing complete metabolic and CNS responses until July 2025, when new CNS lesions developed. He underwent right suboccipital craniotomy followed by postoperative radiation; extracranial disease remained in remission. He later elected hospice care but is alive at manuscript submission (January 2026).

### 2.2. Case 2

**Figure 3:**
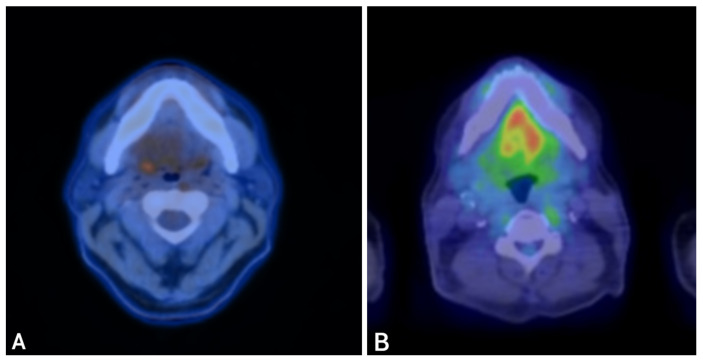
PET-CT imaging before and after fluoropyrimidine-based therapy in Case 2. Legend: Representative PET-CT images demonstrating disease before therapy and response after fluoropyrimidine-based treatment. Pre-treatment (**A**): FDG-avid right cervical lymphadenopathy and mild asymmetric uptake in the right palatine tonsillar region. Post-treatment (**B**): Restaging PET-CT demonstrates resolution of metabolic activity, with decreased enhancement at the primary site and marked reduction in cervical nodal size, consistent with a complete metabolic response.

A 61-year-old never-smoking male with no significant past medical history presented in November 2023 with right-sided tongue and ear pain. CT imaging revealed levels II and III cervical lymphadenopathy. Ultrasound-guided fine-needle aspiration of a right cervical lymph node showed metastatic adenocarcinoma with mucinous features. Immunohistochemistry demonstrated diffuse strong nuclear positivity for CDX2 (Figure 1B,E) and CK20, focal positivity for CK7, and negativity for ER, HER2, SATB2, SOX10, and TTF-1. A staging PET-CT revealed FDG-avid right cervical lymphadenopathy and mild asymmetric uptake in the right palatine tonsillar region, with no evidence of distant metastasis (Figure 3A).

Evaluation in the Head and Neck Oncology Clinic at Mayo Clinic favored a diagnosis of minor salivary gland adenocarcinoma with enteric differentiation and regional nodal metastases, based on histopathologic review of the available tissue and the anatomic distribution of disease, as a direct intraoral biopsy was not obtained. Definitive surgical management with curative intent would have required near-total glossectomy followed by high-dose adjuvant radiotherapy, resulting in substantial and permanent functional morbidity, including severe impairment of speech and swallowing. Although technically feasible, this approach was associated with a high risk of long-term functional decline. Therefore, non-surgical management was prioritized to preserve organ function, in alignment with the patient’s preference for organ preservation.

Given the tumor’s CDX2 positivity, chemotherapy with FOLFOXIRI (leucovorin, 5-FU, oxaliplatin, and irinotecan) was initiated. Soon after starting treatment, the patient experienced a remarkable clinical improvement, allowing discontinuation of around-the-clock oxycodone. Irinotecan was later omitted due to GI toxicity, and chemotherapy continued as modified FOLFOX with dose adjustments.

The patient then transitioned to concurrent chemoradiation, with FOLFOX administered at radiosensitizing doses. Three months after completion of chemoradiation, restaging with PET-CT demonstrated no evidence of active disease, with decreased enhancement at the primary site and reduction in nodal size (Figure 3B). Serial MRIs over subsequent months showed stable post-treatment changes without progression. The patient has now been without evidence of active disease for two years, maintaining good functional status with preserved speech and swallowing.

### 2.3. Case 3

A 48-year-old never-smoker male with no significant past medical history presented in July 2023 with recurrent, refractory epistaxis. ENT evaluation revealed a left nasal cavity mass. Biopsy demonstrated intestinal-type adenocarcinoma positive for CK7, CK20, and diffuse strong nuclear CDX2 (Figure 1C,F), with a CPS of 10 and HER2 2+. CT of the sinuses revealed an extensive left paranasal sinus mass without nodal or distant metastases. At an outside institution, the patient underwent endoscopic craniofacial resection of the anterior cranial fossa, followed by adjuvant proton beam radiation, completed in May 2024.

**Figure 4:**
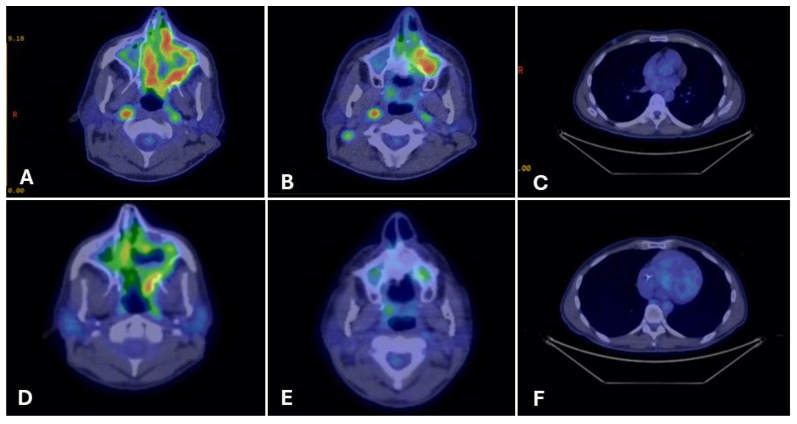
PET-CT imaging before and after fluoropyrimidine-based therapy in Case 3. Legend: Representative PET-CT images show FDG-avid disease at multiple sites before therapy and the response after fluoropyrimidine-based treatment. Pre-treatment (Panels (**A**–**C**)): FDG-avid disease involving the left maxillary sinus mass (**A**), right cervical lymph nodes (**B**), and right pulmonary nodules (**C**). Post-treatment (Panels (**D**–**F**)): Restaging PET-CT images demonstrate remarkable partial metabolic response at all previously involved sites, with reduction in FDG uptake and lesion size (**D**–**F**).

Three months later, surveillance imaging showed local recurrence. Repeat endoscopic resection demonstrated discordant margins, which persisted after a second re-resection. Pembrolizumab was started, but after three doses PET-CT showed progression with FDG-avid sinonasal, orbital, vomer, right cervical nodal, and pulmonary involvement (Figure 4A–C).

The patient was referred for a second opinion to the Head and Neck Oncology Clinic at our institution. He reported persistent sinonasal pressure requiring Norco every five hours and diplopia interfering with daily activities. Given strong CDX2 expression consistent with enteric differentiation, FOLFOXIRI chemotherapy was initiated.

After three months, restaging PET-CT showed markedly decreased sinonasal uptake, improved nodal disease, and near-complete resolution of pulmonary nodules (Figure 4D–F). Clinically, the patient experienced significant pain relief (2/10 from 7/10) and complete resolution of diplopia.

## 3. Discussion

In this series, CDX2 served as a marker of enteric differentiation and was associated with clinical responses to fluoropyrimidine-based therapy across three rare, biologically distinct malignancies. In each case, diffuse strong CDX2 expression indicated an intestinal phenotype and informed the decision to use fluoropyrimidine-based chemotherapy, typically reserved for colorectal cancer. All three patients achieved meaningful metabolic responses, supporting the hypothesis that CDX2 may identify tumors intrinsically sensitive to GI-type regimens regardless of anatomic origin.

These cases suggest that CDX2 expression may merit evaluation as a treatment selection marker in prospective studies, particularly in settings where conventional therapeutic strategies are limited. Case 1 exemplified aggressive variant prostate cancer (AVPC), a subtype associated with poor prognosis and limited responsiveness to androgen-directed therapies. Standard systemic options for AVPC typically include platinum–taxane combinations [27], and cabazitaxel would ordinarily represent the next-line therapy after docetaxel failure [28]. Instead, CDX2 positivity suggested an alternative phenotype-driven vulnerability, and the patient achieved an exceptional and durable extracranial response to fluoropyrimidine-based therapy.

Similarly, Case 2 involved a CDX2-positive minor salivary gland adenocarcinoma, for which no universally accepted systemic standard exists. Historically, multi-agent regimens such as cyclophosphamide–doxorubicin–cisplatin achieve objective response rates in the range of 20–30% with modest durability (median 5–9 months) [29,30]. Randomized data are scarce; in one small phase II trial, vinorelbine–cisplatin improved response over vinorelbine alone but at the cost of increased toxicity [31]. Against this backdrop, the marked radiologic and clinical improvement observed with fluoropyrimidine-based therapy underscores the potential value of CDX2-guided therapy selection in tumor types lacking effective systemic options.

Case 3, a recurrent intestinal-type sinonasal adenocarcinoma, further supports this concept. Systemic therapy for advanced sinonasal malignancies is not standardized and is often extrapolated from regimens used for head and neck squamous cell carcinoma, including pembrolizumab-based combinations [32] and platinum doublets frequently incorporating a taxane [33]. This patient progressed rapidly on pembrolizumab but experienced a pronounced metabolic response and symptomatic improvement with a histology-driven, fluoropyrimidine-based approach. Such responses reinforce the notion that CDX2 positivity reflects a biologic program more aligned with intestinal epithelium than with head and neck carcinoma.

Drawing on existing literature, we hypothesize a unifying conceptual model in which CDX2 expression may reflect a transcriptional program governing enterocyte differentiation and tumor biology. Beyond its diagnostic role, CDX2 has been implicated in modulation of Wnt/β-catenin signaling [34], regulation of genes involved in fluoropyrimidine metabolism, and maintenance of intact p53-mediated apoptotic pathways. Loss of CDX2 in colorectal cancer is associated with poor differentiation and adverse outcomes [35], whereas CDX2-positive tumors generally retain more favorable baseline biology [36]. CDX2-mediated attenuation of proliferative and Wnt-driven signaling may foster a cellular environment more permissive to thymidylate synthase-targeting therapies such as 5-fluorouracil [37,38,39], and preserved tumor suppressor pathways may facilitate robust apoptotic responses to DNA damage [40,41].

However, these mechanistic inferences are derived from prior experimental and clinical studies rather than direct analyses in our cohort. Our case series did not include assessment of thymidylate synthase expression, Wnt pathway activity, or apoptotic signaling, and whether these mechanisms account for the observed clinical responses remains speculative and requires experimental validation. If confirmed, this phenotype-driven vulnerability would support CDX2 as a functional biomarker complementing genomic profiling in rare malignancies with limited therapeutic options.

This study has important limitations that preclude clinical implementation of CDX2-guided therapy selection. First, as a small retrospective case series including only three patients, we cannot establish the predictive value, sensitivity, specificity, or clinical utility of CDX2 as a therapeutic biomarker. The favorable outcomes observed may reflect chance findings, selection bias, patient-specific factors, prior treatments, or other tumor- and patient-specific biological features beyond CDX2 status alone. Consequently, this report is descriptive and hypothesis-generating and should not be interpreted as evidence of a 100% response rate or definitive treatment efficacy. Although formal evaluation of CDX2 sensitivity and specificity in predicting fluoropyrimidine response cannot be determined from this small series, our prior retrospective cohort of 209 CDX2-positive CUP demonstrated an overall response rate to fluoropyrimidine-based therapy of approximately 70% [26], supporting the potential biological relevance of CDX2 expression in guiding therapy. These findings remain hypothesis-generating and warrant prospective validation.

Second, we did not systematically evaluate all CDX2-positive tumors during the study period, nor did we include control comparisons with CDX2-negative tumors or CDX2-positive tumors treated with alternative regimens. Third, the mechanistic model proposed is hypothesis-generating and was not directly tested in these patients. Fourth, heterogeneity in tumor types, prior treatments, disease stage, and response assessment methods further limits generalizability. Most critically, prospective validation in appropriately designed clinical trials is essential before CDX2 status should influence clinical decision-making. Until such validation is available, these observations should be regarded as exploratory and hypothesis-generating rather than practice-changing.

## 4. Conclusions

This case series suggests that CDX2 expression may identify a subset of non-GI tumors with an enteric phenotype that are responsive to fluoropyrimidine-based therapy. Although limited by small sample size and retrospective design, these findings support further prospective evaluation of CDX2 as a potential predictive biomarker to guide phenotype-driven treatment selection across diverse malignancies.

## Data Availability

Data are contained within the article.
